# Differences in the quantitative parameter of lung lesions in dynamic chest FDG PET/CT imaging: framing protocol, reconstruction algorithm, software processing

**DOI:** 10.3389/fonc.2026.1754075

**Published:** 2026-09-18

**Authors:** Yarong Zhang, Xieraili Wumener, Maoqun Zhang, Jiuhui Zhao, Xin Tian, Ying Liang

**Affiliations:** Department of Nuclear Medicine, National Cancer Center/National Clinical Research Center for Cancer/Cancer Hospital & Shenzhen Hospital, Chinese Academy of Medical Sciences and Peking Union Medical College, Shenzhen, China

**Keywords:** BSREM, dPET/CT, framing protocol, K_i_, pitch-in

## Abstract

**Objective:**

This study compares kinetic parameters (K_1_, k_2_, k_3_, K_i_) from dynamic chest PET/CT for lung lesions across different framing protocols, reconstruction algorithms, and software processing, aiming to evaluate quantification consistency and support multi-center standardization.

**Methods:**

This study enrolled 107 patients with 124 lung lesions who underwent 65-min dynamic ^18^F-FDG PET/CT. Dynamic data were compared across three factors: framing protocols (28 frames vs. 39 frames), reconstruction algorithms (the Block Sequential Regularized Expectation Maximization [BSREM] group vs. the third-order Hermite interpolation [Pitch-In] group), and software platforms (the Matlab group vs. the PMOD group). Kinetic parameters were derived based on an irreversible two-tissue compartment model. Subjective image quality was graded by two experts using a 5-point Likert scale.We performed Wilcoxon signed-rank tests with Bonferroni correction, intraclass correlation coefficients (ICCs), Bland–Altman analysis, and *post-hoc* power analysis (n=125) to evaluate statistical significance, consistency, and agreement.

**Results:**

The Pitch-In group achieved scores >3 points and demonstrated significantly superior subjective image quality over the BSREM group (*P* < 0.05), except for comparable lesion discrimination. For kinetic parameter quantification, no statistically significant systematic differences were observed between the Pitch-In group and the BSREM group for K_1_, k_3_, and K_i_ (*P* > 0.05; ICCs: 0.795–0.955), though Pitch-In yielded higher k_2_ values (*P* = 0.003). No significant differences were detected between the Matlab group and the PMOD group for K_1_ and K_i_ (*P* > 0.05), achieving high absolute agreement (ICCs: 0.854 and 0.978; K_i_ bias: 0.000). Between 28 frames and 39 frames protocols, no significant differences were found for all parameters (*P* > 0.05; ICCs: 0.885–0.957), with a minimal K_i_ bias of 0.001 (95% LoA: -0.010 to 0.011).

**Conclusions:**

The primary quantitative endpoint, the K_i_ value, remains highly robust across all evaluated framing protocols, software platforms, and reconstruction algorithms. This strong methodological equivalence facilitates cross-center data harmonization and clinical standardization for multi-center dynamic ^18^F-FDG PET/CT.

## 1 Introduction

^18^F-FDG PET/CT is widely utilized in clinical settings for tumor diagnosis, staging, treatment assessment, and recurrence monitoring, as well as for evaluating inflammation and infection in cardiovascular and neurological diseases (1, 2). In static imaging, patients undergo a distribution period following the injection of ^18^F-FDG, prior to receiving integrated PET and CT scans, thereby generating comprehensive images that simultaneously provide metabolic activity and anatomical structural information (3). In the clinical application of static ^18^F-FDG PET/CT, the Standardized Uptake Values (SUVs) serve as the primary semi-quantitative index to assess radiotracer uptake. SUVs have been extensively utilized to differentiate benign from malignant tumors, evaluate treatment efficacy, and predict prognosis in oncology (4). However, despite their widespread utility, SUVs are influenced by various factors, including scan parameters, equipment variability, and reconstruction algorithms (5). These dependencies introduce variability into the metric, underscoring the critical need for more precise and absolute quantitative metrics to ensure accurate and reliable comparisons.

Dynamic PET/CT (dPET/CT) characterizes the spatio-temporal distribution and metabolic kinetics of radiotracers within the body via continuous, time-resolved acquisition (6). By applying appropriate kinetic modeling, absolute quantitative parameters—including the net influx rate (K_i_), the blood-to-tissue transport constant (K_1_), the tissue-to-blood efflux constant (k_2_), and the phosphorylation rate (k_3_)—can be derived (7, 8). In clinical oncology, K_i_ is the most frequently utilized metric in ^18^F-FDG dPET/CT, providing superior specificity and more rigorous quantification compared to the conventional SUV. Cumulative evidence suggests that K_i_ enhances the accuracy of tumor characterization, facilitating the robust differentiation of benign conditions from malignancies, including primary tumors and involved lymph nodes (7–11). Furthermore, monitoring longitudinal changes in K_i_ provides a powerful imaging biomarker for evaluating therapeutic response (12, 13). Beyond K_i_, other dynamic parameters such as k_2_ and k_3_ have demonstrated significant value in the differential diagnosis of lung cancer (11), while K_1_ and k_2_ have shown substantial efficacy in characterizing metastatic lymph nodes (9).

In recent years, advancements in diagnostic research and therapeutic monitoring have shifted focus toward whole-body dPET/CT, driving increased interest in the clinical application of kinetic modeling (14). The image-derived input function (IDIF) represents the arterial radiotracer concentration (e.g., ^18^F-FDG) measured over time directly from the images. Accurate IDIF estimation is fundamental for the rigorous quantification of tissue tracer uptake. The time framing protocol—referring to the temporal partitioning of dynamic PET acquisitions—is a critical factor in this process. Employing shorter-duration initial frames is essential to capture the rapid peak in tracer activity, thereby ensuring IDIF fidelity (15, 16). Consequently, the design of these protocols directly impacts the IDIF and subsequent kinetic parameters, such as K_i_. A variety of framing protocols coexist in current dPET/CT practices. While existing studies suggest that protocols utilizing 28 frames and 39 frames offer sufficient reliability for parameter estimation (17), emerging evidence emphasizes the intrinsic value of Time-Activity Curve (TAC) morphology in reflecting underlying physiological processes (18). Consequently, indiscriminately reducing the number of frames or overly condensing time intervals may inadvertently sacrifice critical kinetic information. This underscores the imperative of maintaining flexibility in framing protocols within clinical practice to accommodate diverse diagnostic requirements and ensure the mathematical and physiological integrity of kinetic modeling. However, further investigation is warranted to determine the precise impact of varying framing protocols on kinetic parameters.

The enhanced temporal resolution inherent in dPET/CT inevitably leads to increased pixel-level noise in the spatial domain (19). Currently, a universally standardized reconstruction method for dPET/CT remains elusive. While the Block Sequential Regularized Expectation Maximization (BSREM) algorithm—a standard in clinical static PET—performs admirably in conventional scans, its application to dynamic imaging often introduces substantial noise, thereby failing to fully satisfy clinical requirements. Consequently, the development of specialized dynamic reconstruction algorithms has become imperative (20–22). In this context, recent studies have highlighted that the third-order Hermite interpolation (Pitch-In) method significantly outperforms traditional approaches in optimizing dPET/CT image quality (23). However, it remains uncertain whether these advanced reconstruction and denoising techniques, despite improving visual quality, introduce systemic variations in dynamic quantitative parameters.

Furthermore, the resulting reconstructed dPET images are typically processed using various analytical software platforms, such as PMOD software, Matlab software, and the uKinetics software package, to derive dynamic metabolic parameters (9, 17, 24). However, to drive multi-center standardization and ensure the clinical generalizability of findings, it is imperative to investigate whether inherent algorithmic variations across these software tools lead to significant discrepancies in the calculated metabolic indices.

Building upon these considerations, this study systematically evaluates and compares the kinetic parameter values derived from two distinct framing protocols, two reconstruction algorithms, and two different software processing. Our objective is to delineate the impact of these multifaceted factors on kinetic quantification and to evaluate the consistency of various dynamic modeling approaches within the context of multi-center standardization. These findings are intended to provide essential guidance for future research and clinical practice, thereby ensuring the robustness, reliability, and comparability of dPET/CT data across different institutional settings.

## 2 Materials and methods

### 2.1 Data acquisition

This study was conducted with the approval of the Ethics Committee of the Cancer Hospital & Shenzhen Hospital, Chinese Academy of Medical Sciences (KYLH2022-1). All patients refrained from strenuous exercise for 24 h and fasted for at least 6 h prior to the scan (Discovery MI PET/CT, GE Healthcare, Milwaukee, USA). Before the injection of ^18^F-FDG, fasting blood glucose levels were confirmed to be below 8.0 mmol/L. During the scan, patients were positioned supine with both arms raised. A breath-hold chest CT scan was performed first, followed by a whole-body CT scan. The CT parameters were configured as follows: tube voltage of 120 kV, tube current ranging from 10 to 220 mA, pitch of 1.375:1, and noise index of 20. Subsequently, ^18^F-FDG was injected at a dose of 4.44–5.92 MBq/kg through an intravenous catheter, followed by immediate dPET scan acquisition of the chest. DPET scanning sequence was conducted continuously for 65 min.

### 2.2 Framing protocol and image reconstruction

Dynamic PET data were processed using the frame protocol of 28 frames, consisting of 6×10s, 4×30s, 4×60s, and 4×120s, and 10×300s. Attenuation correction was performed using the corresponding CT data. For image reconstruction, the BSREM algorithm was employed with 25 iterations and 2 subsets; images generated via this method were designated as the BSREM group. Additionally, images reconstructed using the Pitch-In method (23) were designated as the Pitch-In group. To evaluate the impact of temporal resolution, the dynamic PET data were also processed according to the frame protocol of 39 frames (6×10s, 8×15s, 6×30s, 6×60s, 4×120s, and 9×300s), with images reconstructed using the BSREM algorithm. The kinetic parameters derived from the 39 frames were then compared with those obtained from the 28 frames to assess the influence of different framing strategies.

### 2.3 Data analysis

#### 2.3.1 Kinetic parameters data analysis

Kinetic parameters, including K_1_, k_2_, k_3_,and K_i_, were obtained based on the two-tissue irreversible compartment model. IDIF was extracted from the ascending aorta by drawing a 10-mm-diameter ROI on six consecutive slices in an image obtained by combining early time frames (0–60s), where the effects of motion and partial volume were less prominent than in the left ventricle. The uptake difference in blood and plasma was not accounted for in this study. In this model, we assumed unidirectional uptake of ^18^F-FDG (i.e., k_4_ = 0), with irreversible trapping in tissue as ^18^F-FDG-6-PO (24). Parametric images of each dynamic scan were generated using Voxel-based analysis. Given a large number of voxels in a whole-body image, the Lawson-Hanson non-negative least squares algorithm was applied to solve a linearized problem instead of the conventional nonlinear one (24). The 3D volume-of-interest (VOI) of each lesion was delineated using the semi-automatic methods with a threshold of 40% SUVmax in ITK-snap software (version 4.0.1). For lesions showing physiological uptake in the periphery, two experienced nuclear medicine physicians manually delineated 3D VOIs on a slice-by-slice basis. Then the segmented VOI was applied to the K_1_, k_2_, k_3_ and K_i_ parametric images to extract the quantitative measurements of each scan. It should be noted that while parametric images were generated voxel-wise to facilitate spatial assessment, all quantitative statistical comparisons in this study were performed using these VOI-averaged metrics. This methodological choice prioritizes the stability and clinical robustness of parameter estimation, as VOI-averaging effectively mitigates the inherent noise associated with individual voxel-level kinetic modeling.

The dynamic data from the 28 frames, the 39 frames, the BSREM group, and the Pitch-In group were analyzed using the aforementioned Matlab software to obtain kinetic parameter values(K_1_, k_2_, k_3_ and K_i_). Subsequently, the dynamic data from the 28 frames were also processed using PMOD software to obtain K_1_ and K_i_ values; this subset was designated as the PMOD group. Correspondingly, the data from the 28 frames analyzed via Matlab software were designated as the Matlab group to evaluate inter-platform consistency.

#### 2.3.2 Subjective image quality assessment

The BSREM images and the Pitch-In images of each subject were anonymized, presented in a random order, and evaluated via a consensus-reading approach by two clinical nuclear medicine experts with more than 3 years of experience. According to the Likert quintile (25), the subjective scores ranging from 1 to 5 of 5 categories were as follows: artifact reduction (ranging from 1 [enormous image artifact] to 5 [no image artifact]), noise suppression (ranging from 1 [enormous image noise] to 5 [no perceivable image noise]), contrast retention (ranging from 1 [hard to distinguish lesion edge] to 5 [very sharp lesion edge]), lesion discrimination (ranging from 1 [difficulty in lesion detection] to 5 [High confidence for small, low uptake lesion]), overall image quality (ranging from 1 [poor overall image quality] to 5 [excellent overall image quality]). Image quality scores of 3 or higher were qualified, indicating that the needs of clinical diagnosis could be met, whereas image quality scores of 1–2 did not meet the needs of clinical diagnosis (15, 26). Any discrepancies between the two experts during the evaluation were resolved through joint discussion until a definitive consensus was reached, which served as the final score.

### 2.4 Statistical analysis

Statistical analysis was performed using SPSS software (version 22.0; SPSS Inc., Chicago, IL, USA) and R software (version 4.5.2; R Foundation for Statistical Computing, Vienna, Austria). The normality of data distribution was assessed using the Shapiro-Wilk test. Given the non-normal distribution, continuous variables were expressed as medians and interquartile ranges (IQRs). Differences between groups were evaluated using the Wilcoxon signed-rank test, with median differences estimated via the Hodges-Lehmann estimator. To control Type I errors across multiple parameter comparisons (K_1_, k_2_, k_3_, and K_i_), P-values were adjusted using the Bonferroni correction. The consistency of kinetic parameters was assessed using the intraclass correlation coefficient (ICC) based on a two-way mixed-effects model with single-measure absolute agreement. Furthermore, effect sizes were calculated via Cliff’s delta, and Bland-Altman analysis was performed. All Bland-Altman plots were generated using R to visualize the agreement between measurements. A *post-hoc* power analysis was conducted based on the sample size (*n* = 125) to detect a small effect size (*d* = 0.3). A two-tailed *P* < 0.05 was considered statistically significant.

## 3 Results

### 3.1 Patient characteristics

From May 2021 to August 2022, patients who provided written informed consent prior to PET/CT imaging and successfully underwent FDG dPET/CT scans of the chest at the hospital were included in the study. In the end, a total of 107 patients were included, comprising 62 males and 45 females, with an average age of 59.89 ± 11.21 years (range: 27–84 years). A total of 124 pulmonary lesions were identified, all of which were confirmed through histopathological biopsy or follow-up CT imaging. Of these, 93 lesions were classified as malignant and 31 as benign. The characteristics of the patients and pulmonary lesions are presented in **Table 1**.

**Table 1:** Characteristics of the patients and lung lesions.

Characteristic	Distribution
**Number of patients**	**107**
Sex (Male)	62 (57.94%)
Age (years)	59.89 ± 11.21 (27~84)
Height (cm)	164.52 ± 9.12 (147~188)
Weight (kg)	63.68 ± 10.77 (44~92)
SUVmax of Lung lesions (malignant/benign)	10.15 ± 6.37 (0.9~26.50)/ 4.56 ± 3.22 (0.9~14.1)
**Pathological types of lung lesions**	**124**
Adenocarcinoma	67 (54.03%)
Squamous cell carcinoma	15 (12.10%)
Small cell carcinoma	5 (4.03%)
Other primary lung malignant tumors	6 (4.84%)
Pulmonary sequestration	1 (0.81%)
Tuberculosis	8 (6.45%)
Nonspecific inflammation	22 (17.74%)

Bold values indicate the total number of patients and the total number of lung lesions included in this study.

### 3.2 Reconstruction algorithm image analysis and parameters evaluation

**Table 2** presents the qualitative visual analysis of images reconstructed using the BSREM and Pitch-In methods in dPET/CT. The Pitch-In group achieved image quality scores exceeding 3 points across all five evaluated categories (artifact reduction, noise suppression, contrast preservation, lesion identification, and overall quality), which was deemed sufficient to meet clinical requirements. **Table 2** shows that there was no significant difference between the BSREM group and the Pitch-In group in terms of lesion discrimination of image quality (P>0.05). There were significant differences (P<0.05) in other four categories of image quality between Pitch-In group images and BSREM group images, and the former was superior to the latter (**Figure 1**). In conclusion, the image quality of the Pitch-In reconstruction was superior to that of the BSREM reconstruction.

**Table 2:** Qualitative analysis results of the BSREM group and the Pitch-In group images.

Category	BSREM group	Pitch-in group	*P* value
Artifact reduction	3.15 ± 1.00	4.22 ± 0.81	<0.01*
Noise suppression	2.78 ± 1.10	3.98 ± 0.86	<0.01*
Contrast retention	3.23 ± 1.02	4.38 ± 0.63	<0.01*
Lesion discrimination	4.19 ± 0.70	4.31 ± 0.73	0.10
Overall image quality	3.07 ± 1.16	4.14 ± 0.81	<0.01*

*stands for statistically significant difference.

**Figure 1:**
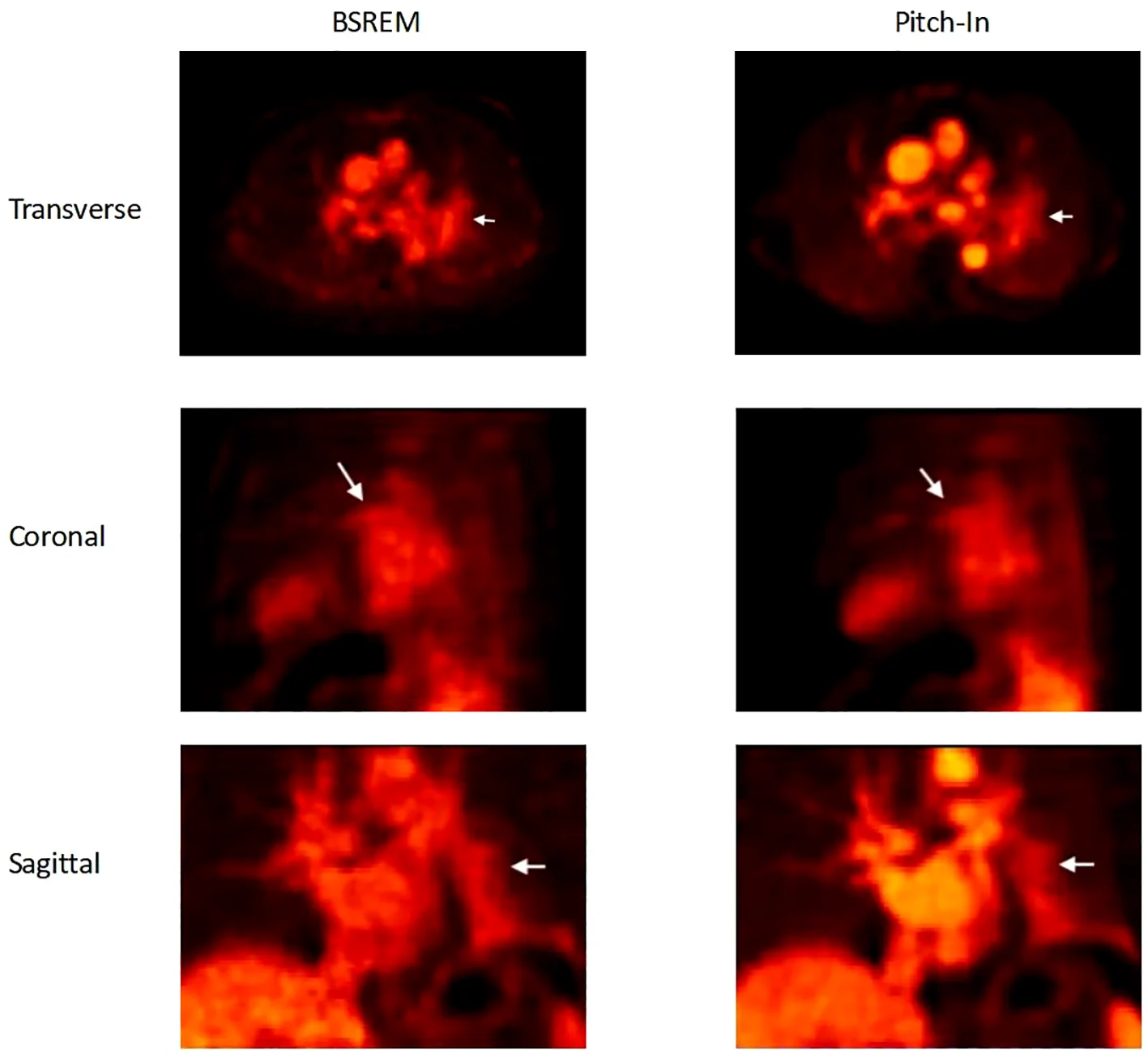
Representative pitch-in images and BSREM images in different planes (the transverse plane, coronal plane and sagittal plane). Compared to BSREM images, Pitch-In images had good quality and displayed visual differences between the two images.

A methodological comparison of kinetic parameters (K_1_, k_2_, k_3_, and K_i_) derived from the BSREM and Pitch-In reconstruction algorithms was conducted, with comprehensive statistical data summarized in **Table 3**. For the K_1_ parameter, no statistically significant difference was detected between the two algorithms (adjusted *P* > 0.05, raw *P* = 0.094; 95% CI of the difference: -0.013 to 0.001), showing a negligible effect size (Cliff’s Delta = -0.033, magnitude 1). The algorithms demonstrated good inter-algorithm reliability with a single-measure absolute agreement ICC of 0.795 (95% CI: 0.720–0.852), while the Bland-Altman plot (**Figure 2**) confirmed a minimal systematic bias (mean difference = -0.000) and narrow limits of agreement (95% LoA: -0.231 to 0.230).

**Table 3:** Comparison of dynamic metabolic parameters between BSREM and pitch-in reconstruction algorithms.

Kinetic parameter	BSREM group [med (IQR)]	Pitch-in group [Med (IQR)]	*P* value^a^	Adjusted *P* value^b^	Median diff (95% CI)^c^	Cliff's δ	ICC (95% CI)^d^
K_1_ (ml/g/min)	0.1487 (0.1036–0.2177)	0.1511 (0.1081–0.2295)	0.094	0.377	-0.0056 (-0.0134, 0.0009)	-0.033	0.795 (0.720–0.852)
k_2_ (min^-1^)	0.3304 (0.1748–0.5520)	0.3686 (0.2029–0.5642)	0.003*	0.011*	-0.0431 (-0.0697, -0.0163)	-0.078	0.700 (0.599–0.780)
k_3_ (min^-1^)	0.0586 (0.0297–0.0792)	0.0589 (0.0283–0.0849)	0.088	0.351	-0.0025 (-0.0053, 0.0004)	-0.018	0.864 (0.811–0.902)
K_i_ (ml/g/min)	0.0232 (0.0108–0.0358)	0.0231 (0.0100–0.0352)	0.066	0.262	0.0005 (0.0000–0.0011)	0.020	0.955 (0.937–0.968)

^a^ Calculated using the Wilcoxon signed-rank test. ^b^P-values adjusted using the Bonferroni correction for multiple parameter comparisons. ^c^Estimated using the Hodges-Lehmann method. ^d^Intraclass correlation coefficient (Two-way mixed, absolute agreement). Med, median; IQR, interquartile range; CI, confidence interval.

*stands for statistically significant difference.

**Figure 2:**
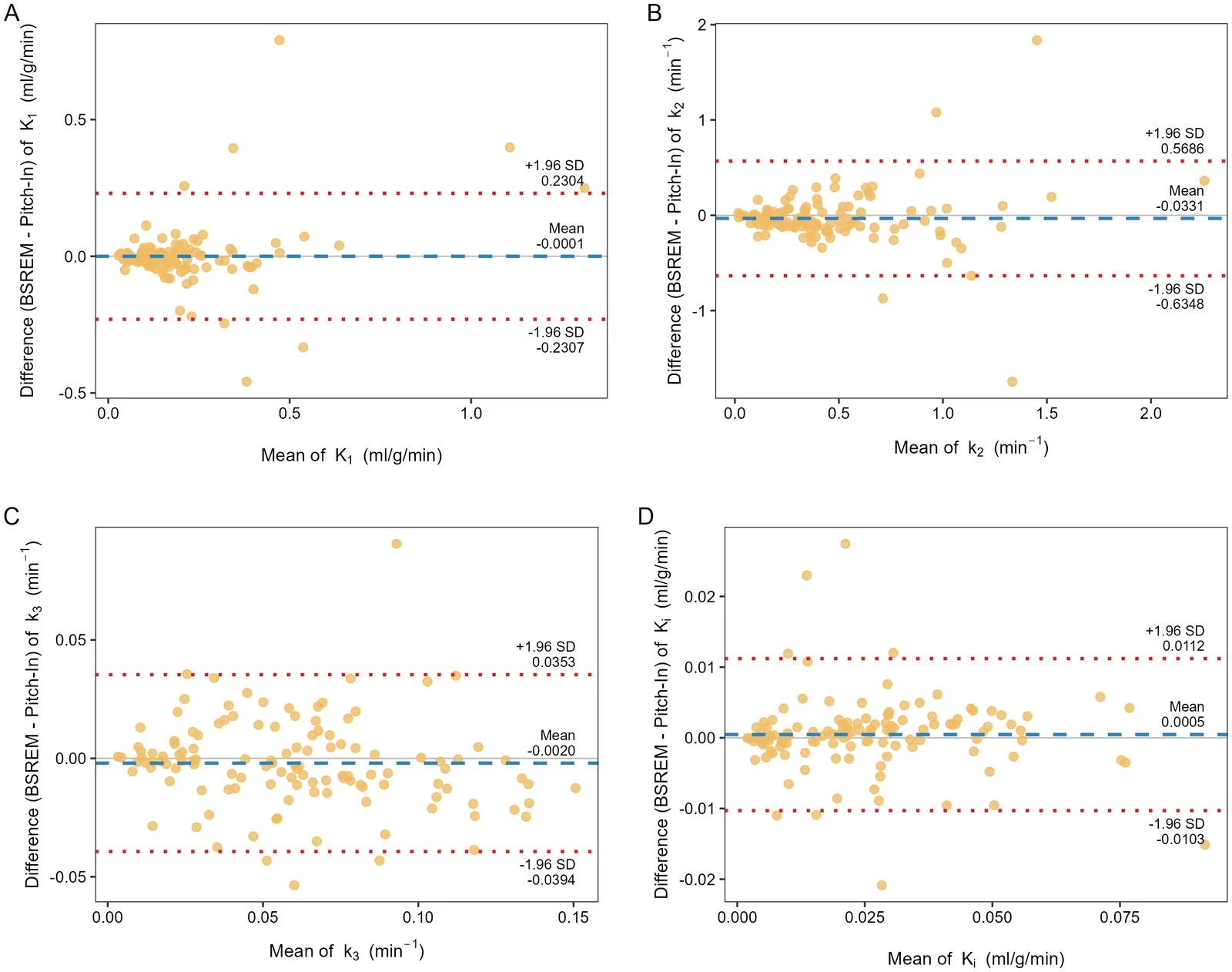
Bland–Altman analysis of kinetic parameters between different reconstruction algorithms. **(A)** Comparison of K_1_ values. **(B)** Comparison of k_2_ values. **(C)** Comparison of k_3_ values. **(D)** Comparison of K_i_ values.

Similarly, no significant differences were observed for the k_3_ and K_i_ parameters(both adjusted *P* > 0.05; raw *P* = 0.088 and 0.066, respectively), with both demonstrating negligible effect sizes (magnitude 1). High to excellent consistency was achieved between the two algorithms, as indicated by ICC values of 0.864 for k_3_ and 0.955 for K_i_. Their absolute agreements were tightly bounded, as illustrated by the Bland-Altman plots (**Figure 2**), showing mean differences close to zero and narrow 95% LoA intervals (k_3_ 95% LoA: -0.039 to 0.035; K_i_ 95% LoA: -0.010 to 0.011).Additionally, a *post-hoc* power analysis (*n* = 125) verified that the study achieved a 91.4% power to detect a small effect size (*d* = 0.300).

In contrast, a statistically significant difference was sustained for the k_2_ parameter (raw *P* = 0.003, adjusted P = 0.011), where the Pitch-In algorithm yielded systematically higher values than the BSREM algorithm (95% CI of the difference: -0.070 to -0.016). Although the relative reliability remained moderate-to-good (ICC = 0.700, 95% CI: 0.599–0.780) with a small effect size (magnitude 1), the Bland-Altman plot (**Figure 2**) revealed a noticeable systematic bias (mean difference = -0.033) and substantially wider limits of agreement (95% LoA: -0.635 to 0.569), indicating lower absolute interchangeability between the two algorithms for k_2_ quantification.

### 3.3 Software processing parameters evaluation

A methodological comparison of kinetic parameters (K_i_ and K_1_) derived from the Matlab and PMOD groups was conducted, with comprehensive statistical data summarized in **Table 4**. For both parameters, no statistically significant differences were detected between the two software workflows (adjusted *P* = 0.225, raw *P* = 0.113 for K_i_; adjusted *P* = 0.368, raw *P* = 0.184 for K_1_), with both demonstrating negligible effect sizes (Cliff’s Delta = 0.018 for K_i_, -0.011 for K_1_). High to excellent consistency was achieved between the two groups, as indicated by a single-measure absolute agreement ICC of 0.978 (95% CI: 0.969–0.985) for K_i_ and 0.854 (95% CI: 0.798–0.896) for K_1_.

**Table 4:** Analysis of dynamic metabolic parameter values in Matlab group and PMOD group.

Kinetic parameter	Matlab group [med (IQR)]	PMOD group [med (IQR)]	*P* value^a^	Adjusted *P* value^b^	Median diff (95% CI)^c^	Cliff's δ	ICC (95% CI)^d^
**K_1_ (ml/g/min)**	0.1487 (0.1036–0.2177)	0.1484 (0.1069–0.2119)	0.184	0.368	-0.0029 (-0.0076, 0.0015)	-0.011	0.854 (0.798–0.896)
**K_i_ (ml/g/min)**	0.0232 (0.0108–0.0358)	0.0238 (0.0101–0.0351)	0.113	0.225	0.0003 (-0.0001, 0.0008)	0.018	0.978 (0.969–0.985)

^a^ Calculated using the Wilcoxon signed-rank test. ^b^P-values adjusted using the Bonferroni correction for multiple parameter comparisons. ^c^Estimated using the Hodges-Lehmann method. ^d^Intraclass correlation coefficient (Two-way mixed, absolute agreement). Med, median; IQR, interquartile range; CI, confidence interval.

*stands for statistically significant difference.

Bland-Altman analysis (**Figure 3**) confirmed high absolute agreement between the Matlab and PMOD groups. For K_i_, the mean bias was 0.000, with 95% LoA ranging from -0.007 to 0.008. For K_1_, a minimal systematic bias of 0.007 was observed, bounded by 95% LoA intervals of -0.185 to 0.199. This underscores that transitioning between the Matlab and PMOD workflows preserved the absolute quantitative integrity of kinetic parameter estimation.

**Figure 3:**
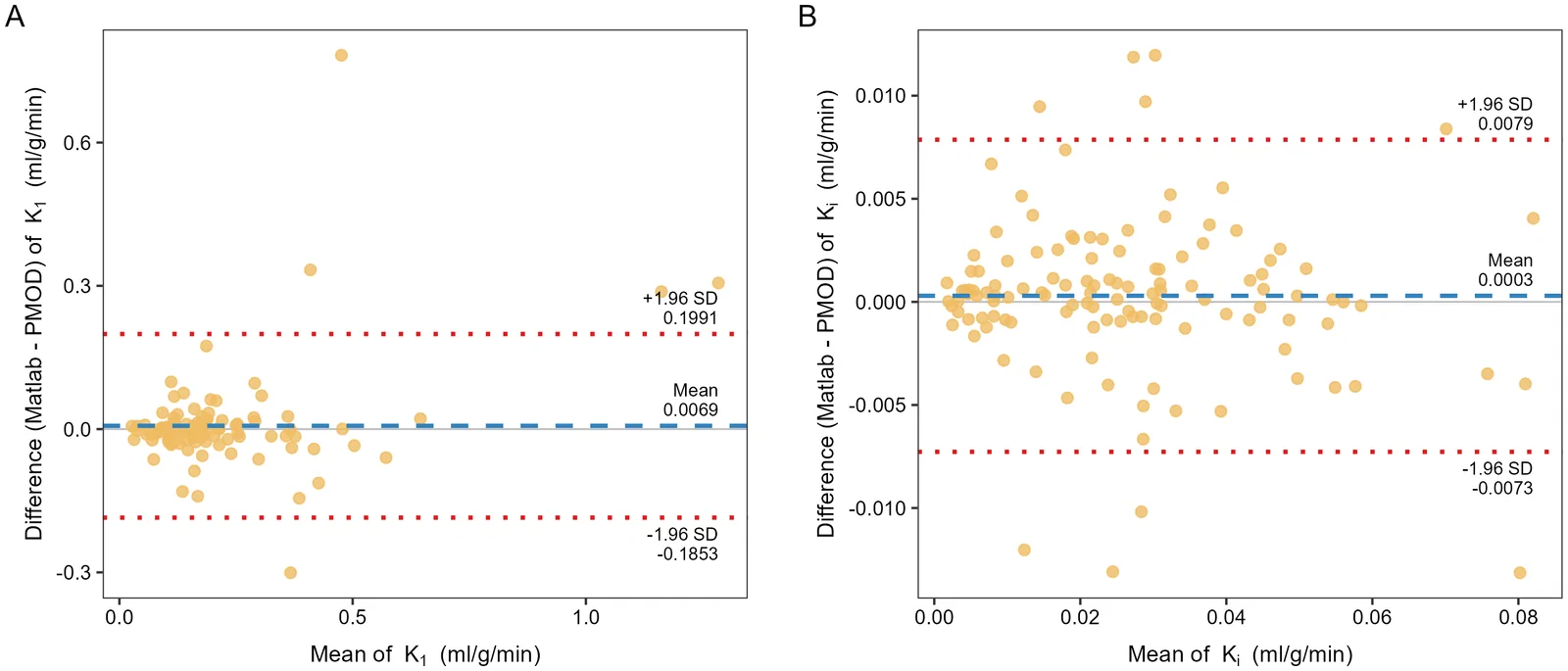
Bland–Altman analysis of kinetic parameters between Matlab group and PMOD group. **(A)** Comparison of K_1_ values. **(B)** Comparison of K_i_ values.

### 3.4 Framing protocol parameters evaluation

A methodological comparison of kinetic parameters (K_1_, k_2_, k_3_, and K_i_) derived from different framing protocols was conducted, with comprehensive statistical data summarized in **Table 5**. For all evaluated parameters, no statistically significant differences were detected between the 28 frames and 39 frames protocols (all adjusted *P* > 0.05; raw *P* = 0.835 for K_1_, 0.505 for k_2_, 0.929 for k_3_, and 0.131 for K_i_), with individual parameters demonstrating distinct negligible effect sizes (Cliff’s Delta: -0.006 for K_1_, -0.027 for k_2_, 0.011 for k_3_, and 0.030 for K_i_). High to excellent consistency was achieved between the two groups, as indicated by single-measure absolute agreement ICC values of 0.895 (95% CI: 0.854–0.925) for K_1_, 0.885 (95% CI: 0.841–0.918) for k_2_, 0.925 (95% CI: 0.895–0.947) for k_3_, and 0.957 (95% CI: 0.939–0.970) for K_i_. The corresponding Bland-Altman plots (**Figure 4**) further substantiated this robust absolute agreement by revealing minimal systematic evaluation biases across all endpoints. Specifically, the mean difference and 95% LoA intervals were 0.004 (-0.177 to 0.186) for K_1_, -0.019 (-0.407 to 0.370) for k_2_, 0.001 (-0.025 to 0.027) for k_3_, and 0.001 (-0.010 to 0.011) for the primary endpoint K_i_. Consequently, modifying the framing setup from 39 frames to 28 frames introduced no statistically tangible penalty to the absolute quantitative integrity of kinetic modeling.

**Table 5:** Analysis of dynamic metabolic parameter values in 28 frames and 39 frames.

Kinetic parameter	28 frames [med (IQR)]	39 frames [med (IQR)]	*P* value^a^	Adjusted *P* value^b^	Median diff (95% CI)^c^	Cliff's δ	ICC (95% CI)^d^
K_1_ (ml/g/min)	0.1487 (0.1036–0.2177)	0.1465 (0.1038–0.2127)	0.835	1.000	0.0004 (-0.0030, 0.0039)	-0.006	0.895 (0.854–0.925)
k_2_ (min^-1^)	0.3304 (0.1748–0.5520)	0.3305 (0.1823–0.5870)	0.505	1.000	-0.0025 (-0.0098, 0.0066)	-0.027	0.885 (0.841–0.918)
k_3_ (min^-1^)	0.0586 (0.0297–0.0792)	0.0578 (0.0273–0.0797)	0.929	1.000	-0.0001 (-0.0013, 0.0014)	0.011	0.925 (0.895–0.947)
K_i_ (ml/g/min)	0.0232 (0.0108–0.0358)	0.0231 (0.0100–0.0352)	0.131	0.523	0.0003 (-0.0001, 0.0007)	0.030	0.957 (0.939–0.970)

^a^ Calculated using the Wilcoxon signed-rank test. ^b^P-values adjusted using the Bonferroni correction for multiple parameter comparisons. ^c^Estimated using the Hodges-Lehmann method. ^d^Intraclass correlation coefficient (Two-way mixed, absolute agreement). Med, median; IQR, interquartile range; CI, confidence interval.

*stands for statistically significant difference.

**Figure 4:**
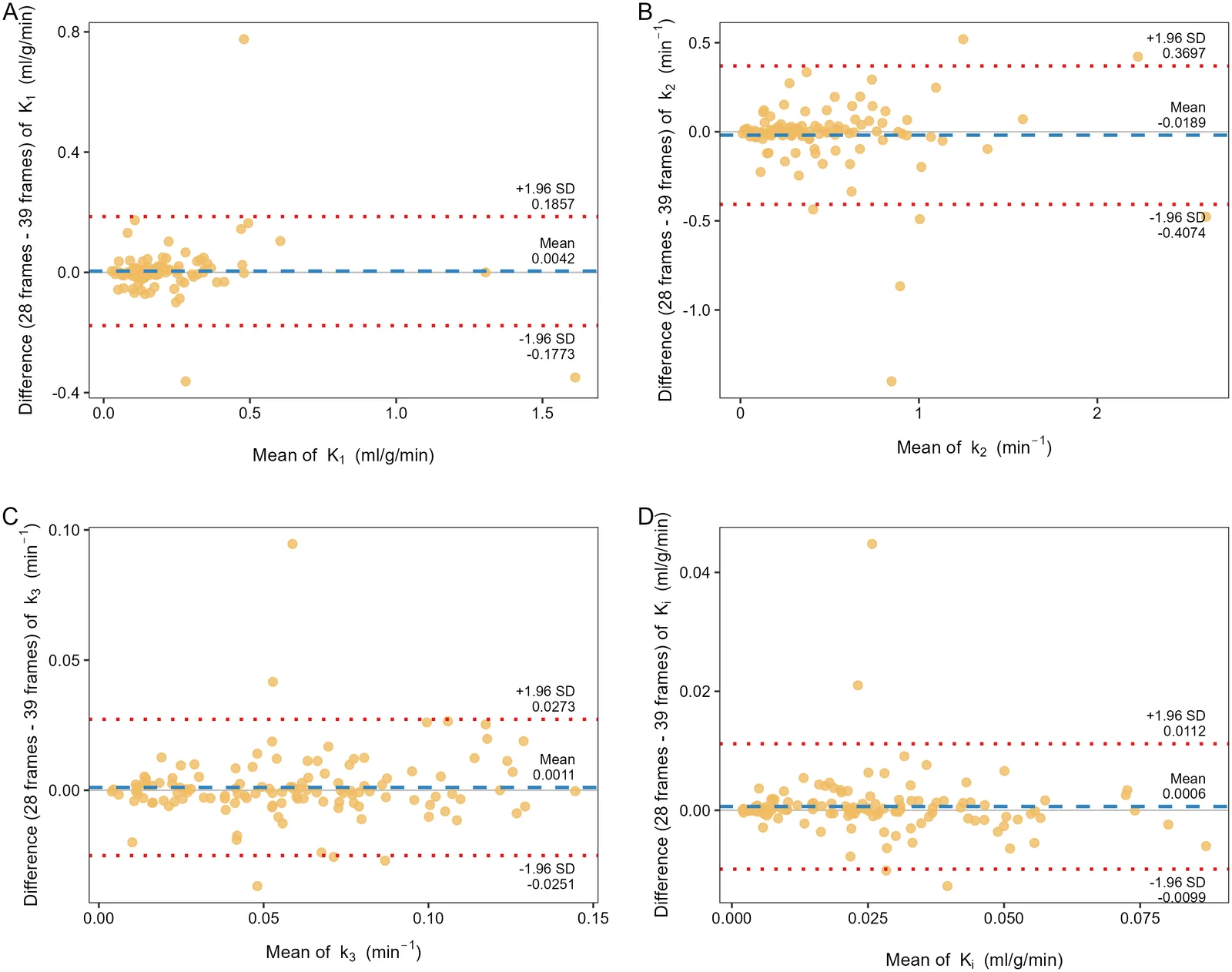
Bland–Altman analysis of kinetic parameters between 28 frames and 39 frames. **(A)** Comparison of K_1_ values. **(B)** Comparison of k_2_ values. **(C)** Comparison of k_3_ values. **(D)** Comparison of K_i_ values.

## 4 Discussion

Currently, several commercial software packages are available for kinetic parameter estimation (14), with PMOD being one of the most widely utilized platforms due to its comprehensive library of standard compartment models. Through a rigorous quantitative comparison, our study demonstrated that the Matlab-based workflow yielded K_i_ and K_1_ estimates highly comparable to those obtained from PMOD, as highlighted by the near-perfect absolute agreement ICC of 0.978 for K_i_. Crucially, this exceptional numerical equivalence firmly reinforces the diagnostic reliability of Matlab-derived K_i_ values, which have been successfully implemented in clinical practice to distinguish between benign and malignant mediastinal and hilar lymph nodes. Beyond confirming this foundational reliability for primary endpoints, a distinct practical advantage of our Matlab platform lies in its seamless efficiency for high-throughput, customized batch processing. While clinical users often limit routines in standard PMOD configurations to the primary operational endpoints (K_i_ and K_1_) to balance workflow efficiency, the Matlab script permits the simultaneous, automated extraction of full-suite micro-parameters—specifically k_2_ and k_3_—without additional software overhead (9). Integrating these multi-parametric metrics permits a more nuanced and direct mathematical characterization of underlying pathophysiological processes, wherein K_1_ delineates the plasma-to-tissue radiotracer transport rate. Given that recent evidence underscores the pivotal role of K_1_, k_2_, and k_3_ in the detection, differential diagnosis, and treatment response evaluation within clinical oncology (27–30), the clinical relevance of these incremental parameters is substantial. Consequently, while standard software workflows can sometimes constrain the routine clinical throughput of advanced multi-parametric scanning, our Matlab-based framework provides a highly accessible, comprehensive, and high-efficiency alternative for expansive dynamic PET applications. It is important to note that our comparative analysis with PMOD was primarily centered on K_i_ and K_1_, as these serve as the standard operational endpoints in our current clinical workflow. The absence of a systematic comparison for k_2_ and k_3_ reflects this study-specific workflow optimization rather than an intrinsic limitation of the PMOD software, which is well-established for comprehensive compartmental modeling.

Although high-temporal-resolution (HTR) dynamic PET (dPET) imaging can capture rapid tracer kinetics and provide precise hemodynamic information (20, 21), its clinical adoption has historically been hindered by poor signal-to-noise ratios (SNR) (14).Consequently, conventional protocols often resort to medium temporal resolutions (e.g., 10–40 s/frame) to balance timing precision against image noise. Overcoming this trade-off requires advanced reconstruction algorithms specifically tailored for HTR frameworks (31, 32). Chen et al. found that Pitch-In, compared to currently known advanced methods, is a powerful denoising algorithm with robust performance in terms of imaging clarity, accuracy, and tissue contrast resolution, making it more stable and clinically feasible (23). Our study verified that Pitch-In processing markedly enhances image quality over traditional BSREM reconstruction. More importantly, this substantial noise reduction was achieved without compromising the quantitative accuracy of macro-parameters, as evidenced by the excellent absolute agreement in K_i_ values. Interestingly, a parameter-specific divergence emerged within this high-performance framework, wherein the Pitch-In algorithm yielded systematically higher k_2_ values and noticeably wider limits of agreement compared to BSREM. This discrepancy may be attributed to the distinct mathematical mechanisms through which each algorithm mitigates the severe noise propagating into low-count data. Consequently, while the integration of advanced reconstruction algorithms ensures negligible variations in K_i_ quantification, maintaining strict methodological consistency remains paramount when clinical investigations focus on k_2_ kinetics.

At present, dPET scanning uses various framing protocols, which renders cross-center standardization and data comparison highly challenging. Therefore, it is imperative to evaluate potential variations in kinetic parameter estimations across different framing frameworks to facilitate their multicenter clinical translation, particularly regarding K_i_ values for therapeutic response evaluation. Zhang et al. found that the kinetic parameters K_i_ and K_1_ values of the dynamic framing protocol at 29 frames are sufficient for application in oncology compared to 100 frames, and do not require protocols with more frames (17). Consistently, our study demonstrated that dividing the dPET/CT scan data of the chest into 28 frames and 39 frames exerted no significant impact on K_i_ values, confirming that the findings from both studies were highly aligned. Meanwhile, this study also revealed that using fewer framing protocols did not affect the remaining parameter values (K_1_, k_2_, k_3_), as evidenced by the high-to-excellent absolute agreement ICC values (ranging from 0.885 to 0.957). Crucially, the statistical equivalence of K_i_ between the 28 frames and 39 frames protocols underscores that a more streamlined framing setup can be widely adopted without sacrificing quantitative accuracy. By significantly lowering the entry barrier for dynamic scanning and reducing computational overhead, these findings provide a solid methodological foundation for harmonizing dPET imaging protocols across different imaging sites. Consequently, our findings indicated that establishing parameter equivalence across varied temporal framing configurations ensures the absolute quantitative integrity of dynamic metabolic assessment, thereby substantially facilitating large-scale multicenter clinical trials and collaborative research.

In summary, our study successfully validated a custom Matlab-based framework that achieves high numerical equivalence to the gold-standard PMOD for K_i_ and K_1_, while enabling cost-effective batch extraction of k_2_ and k_3_. Furthermore, the robust parameter consistency demonstrated across both varied temporal framing configurations and advanced reconstruction algorithms (Pitch-In vs. BSREM) firmly preserves the absolute quantitative integrity of primary K_i_ measurements. By defining these boundaries of parameter stability, this work provides a dependable methodological foundation to standardize dynamic PET protocols and facilitate future cross-center collaborative research.

It must be acknowledged that the current study primarily employed an indirect parameter estimation approach based on a 2-tissue irreversible kinetic model to assess the robustness of K_i_. While this model is well-validated for specific clinical research protocols, it differs from the broader class of clinically deployed graphical analysis methods, such as standard Patlak, generalized Patlak, and direct 4D parametric reconstruction approaches (33–38). Modern commercial clinical platforms (e.g., Siemens Flow and United Imaging uKinetics) have widely integrated these graphical analysis tools to support both multi-bed and total-body dynamic PET parametric imaging (33, 35, 37, 38). Furthermore, our study assumes irreversible FDG kinetics (k_4_ = 0), a simplification that neglects potential tracer reversibility (k_4_ > 0) observed in certain organ systems and oncologic tumors, which may lead to systematic underestimations of k_3_ and K_i_ (4, 37–39). Although a 2-tissue irreversible kinetic model remains a pragmatic compromise between physiological precision and the stability required for routine clinical practice, quantitative PET has evolved from simplified metrics like the standardized uptake value toward more robust kinetic and parametric imaging workflows (4). Therefore, our findings regarding K_i_ stability should be interpreted specifically within the context of compartmental-model-derived parameters and should not be extrapolated to the wider range of clinically deployed Patlak-based estimation tools. Future efforts should prioritize the integration of advanced graphical analysis methods—such as generalized Patlak, which offers enhanced robustness in mitigating tracer reversibility biases—to better account for uptake heterogeneity across diverse technical variations (4, 37, 38).

The main limitations of our study are as follows: (i) This study focused exclusively on single-bed dynamic chest PET acquisitions, which may not fully represent the complexities of modern whole-body oncologic workflows. Given that kinetic parameter stability may differ under multi-bed transitions, axial sensitivity variations, and long-AFOV acquisitions, our findings should be interpreted as a reproducible baseline for single-bed acquisitions. Further studies are required to validate these findings within the broader context of multi-bed and long-AFOV total-body PET systems, which represent the current clinical standard. (ii) The framing protocols and reconstruction methods utilized herein were not based on official recommendations or guidelines, and only two specific configurations were selected for analysis. Therefore, the potential impact of other alternative framing schemes and reconstruction algorithms on kinetic parameter values remains to be further investigated. (iii) Finally, this study focused on the methodological robustness of kinetic parameters and did not directly correlate these values with their diagnostic value in identifying malignant versus benign lesions. Future studies, involving larger datasets, will be required to explicitly test whether different technical workflows (i.e., time framing, reconstruction methods, and software tools) yield identical diagnostic performance.

## 5 Conclusion

In conclusion, this study demonstrates that the primary quantitative endpoint, the K_i_ value, showed no significant differences across the specific temporal framing setups, analytical software, or image reconstruction algorithms evaluated in this work. These results suggest a high methodological equivalence among these configurations, which is expected to facilitate cross-center data harmonization and potentially accelerate the clinical translation of multicenter dynamic ^18^F-FDG PET/CT imaging.

## Data Availability

The raw data supporting the conclusions of this article will be made available by the authors, without undue reservation.

## References

[1] Anand S Singh H Dash A . Clinical applications of PET and PET-CT. Med J Armed Forces India. (2009) 65:353–8. doi: 10.1016/S0377-1237(09)80099-3 27408291 PMC4921358

[2] Dimitrakopoulou-Strauss A . PET-based molecular imaging in personalized oncology: potential of the assessment of therapeutic outcome. Future Oncol. (2015) 11:1083–91. doi: 10.2217/fon.15.28 25804123

[3] Boellaard R Delgado-Bolton R Oyen WJG Giammarile F Tatsch K Eschner W . FDG PET/CT: EANM procedure guidelines for tumour imaging: version 2.0. Eur J Nucl Med Mol Imaging. (2015) 42:328–54. doi: 10.1007/s00259-014-2961-x 25452219 PMC4315529

[4] Huang SC . Anatomy of SUV. Standardized uptake value. Nucl Med Biol. (2000) 27:643. doi: 10.1016/S0969-8051(00)00155-4 11091106

[5] Tomasi G Turkheimer F Aboagye E . Importance of quantification for the analysis of PET data in oncology: review of current methods and trends for the future. Mol Imaging Biol. (2012) 14:131–46. doi: 10.1007/s11307-011-0514-2 21842339

[6] Carson RE . 6 Tracer Kinetic Modeling in PET. In: Positron Emission Tomography. Medical Radiology. Berlin, Heidelberg: Springer. (2006) p. 133–161. doi: 10.1007/1-84628-007-9_6

[7] Takesh M . The potential benefit by application of kinetic analysis of PET in the clinical oncology. ISRN Oncol. (2012) 2012:1–9. doi: 10.5402/2012/349351 23326682 PMC3541563

[8] Rahmim A Lodge MA Karakatsanis NA Panin VY Zhou Y McMillan A . Dynamic whole-body PET imaging: principles, potentials and applications. Eur J Nucl Med Mol Imaging. (2019) 46:501–18. doi: 10.1007/s00259-018-4153-6 30269154

[9] Wumener X Zhang Y Wang Z Zhang M Zang Z Huang B . Dynamic FDG-PET imaging for differentiating metastatic from non-metastatic lymph nodes of lung cancer. Front Oncol. (2022) 12:1005924. doi: 10.3389/fonc.2022.1005924 36439506 PMC9686335

[10] Gupta N Gill H Graeber G Bishop H Hurst J Stephens T . Dynamic positron emission tomography with F-18 fluorodeoxyglucose imaging in differentiation of benign from Malignant lung/mediastinal lesions. Chest. (1998) 114:1105–11. doi: 10.1378/chest.114.4.1105 9792584

[11] Wumener X Zhang Y Zang Z Du F Ye X Zhang M . The value of dynamic FDG PET/CT in the differential diagnosis of lung cancer and predicting EGFR mutations. BMC Pulm Med. (2024) 24:227. doi: 10.1186/s12890-024-02997-9 38730287 PMC11088023

[12] Freedman N Sundaram S Kurdziel K Carrasquillo J Whatley M Carson J . Comparison of SUV and Patlak slope for monitoring of cancer therapy using serial PET scans. Eur J Nucl Med Mol Imaging. (2003) 30:46–53. doi: 10.1007/s00259-002-0981-4 12483409

[13] Cheebsumon P Velasquez LM Hoekstra CJ Hayes W Kloet RW Hoetjes NJ . Measuring response to therapy using FDG PET: semi-quantitative and full kinetic analysis. Eur J Nucl Med Mol Imaging. (2011) 38:832–42. doi: 10.1007/s00259-010-1705-9 21210109 PMC3070082

[14] Wang G Rahmim A Gunn RN . PET parametric imaging: past, present, and future. IEEE Trans Radiat Plasma Med Sci. (2020) 4:663–75. doi: 10.1109/TRPMS.2020.3025086 33763624 PMC7983029

[15] Huang Z Wu Y Fu F Meng N Gu F Wu Q . Parametric image generation with the uEXPLORER total-body PET/CT system through deep learning. Eur J Nucl Med Mol Imaging. (2022) 49:2482–92. doi: 10.1007/s00259-022-05731-x 35312030

[16] Zhang X Xie Z Berg E Judenhofer MS Liu W Xu T . Total-body dynamic reconstruction and parametric imaging on the uEXPLORER. J Nucl Med. (2020) 61:285–91. doi: 10.2967/jnumed.119.230565 31302637 PMC8801950

[17] Ye Q Zeng H Zhao Y Zhang W Dong Y Fan W . Framing protocol optimization in oncological Patlak parametric imaging with uKinetics. EJNMMI Phys. (2023) 10:54. doi: 10.1186/s40658-023-00577-0 37698773 PMC10497476

[18] Yao Z Wang Y Wu Y Zhou J Dang N Wang M . Leveraging machine learning with dynamic 18F-FDG PET/CT: integrating metabolic and flow features for lung cancer differential diagnosis. Eur J Nucl Med Mol Imaging. (2025) 52:3807–19. doi: 10.1007/s00259-025-07231-0 40183949 PMC12316803

[19] Kotasidis FA Tsoumpas C Rahmim A . Advanced kinetic modelling strategies: towards adoption in clinical PET imaging. Clin Transl Imaging. (2014) 2:219–37. doi: 10.1007/s40336-014-0069-830311153

[20] Wang G Sarkar S Kim E Badawi R . Time-varying kinetic modeling of high temporal-resolution dynamic 18F-FDG PET data for multiparametric imaging. J Nucl Med. (2018) 59:1412–7. doi: 10.2967/jnumed.117.202359

[21] Wang G Spencer B Sarkar S Shi H Chen S Hu P . Quantification of glucose transport using high temporal resolution dynamic PET imaging. J Nucl Med. (2019) 60:1332–7. doi: 10.2967/jnumed.118.219805

[22] Zuo Y Badawi RD Foster CC Smith T Lopez JE Wang G . Multiparametric cardiac ^18^ F-FDG PET in humans: kinetic model selection and identifiability analysis. IEEE Trans Radiat Plasma Med Sci. (2020) 4:759–67. doi: 10.1109/TRPMS.2020.3031274 33778234 PMC7996630

[23] Chen Z Wu Y Zhang N Sun T Shen Y Zheng H . High temporal resolution total-body dynamic PET imaging based on pixel-level time-activity curve correction. IEEE Trans BioMed Eng. (2022) 69:3520–31. doi: 10.1109/TBME.2022.3176097 35594213

[24] Patlak CS Blasberg RG Fenstermacher JD . Graphical evaluation of blood-to-brain transfer constants from multiple-time uptake data. J Cereb Blood Flow Metab. (1983) 3:1–7. doi: 10.1038/jcbfm.1983.1 6822610

[25] Van Sluis J Boellaard R Somasundaram A Van Snick PH Borra RJH Dierckx RAJO . Image quality and semiquantitative measurements on the Biograph Vision PET/CT system: initial experiences and comparison with the Biograph mCT. J Nucl Med. (2020) 61:129–35. doi: 10.2967/jnumed.119.227801 31253742

[26] Wang H Wu Y Huang Z Li Z Zhang N Fu F . Deep learning–based dynamic PET parametric Ki image generation from lung static PET. Eur Radiol. (2023) 33:3522–33. doi: 10.1007/s00330-022-09237-w 36399164

[27] Dunnwald LK Doot RK Specht JM Gralow JR Ellis GK Livingston RB . PET tumor metabolism in locally advanced breast cancer patients undergoing neoadjuvant chemotherapy: value of static versus kinetic measures of fluorodeoxyglucose uptake. Clin Cancer Res. (2011) 17:2400–9. doi: 10.1158/1078-0432.CCR-10-2649 21364034 PMC3086719

[28] Nishimura M Tamaki N Matsushima S Kiba M Kotani T Bamba C . Dynamic whole-body 18F-FDG PET for differentiating abnormal lesions from physiological uptake. Eur J Nucl Med Mol Imaging. (2020) 47:2293–300. doi: 10.1007/s00259-020-04726-w 32189027

[29] Sachpekidis C Hassel JC Kopp-Schneider A Haberkorn U Dimitrakopoulou-Strauss A . Quantitative dynamic 18F-FDG PET/CT in survival prediction of metastatic melanoma under PD-1 inhibitors. Cancers. (2021) 13:1019. doi: 10.3390/cancers13051019 33804417 PMC7957728

[30] De Prost N Feng Y Wellman T Tucci MR Costa EL Musch G . ^18^ F-FDG kinetics parameters depend on the mechanism of injury in early experimental acute respiratory distress syndrome. J Nucl Med. (2014) 55:1871–7. doi: 10.2967/jnumed.114.140962 25286924 PMC4258066

[31] Wang G . High temporal-resolution dynamic PET image reconstruction using a new spatiotemporal kernel method. IEEE Trans Med Imaging. (2019) 38:664–74. doi: 10.1109/TMI.2018.2869868 30222553 PMC6422751

[32] Sun H Peng L Zhang H He Y Cao S Lu L . Dynamic PET image denoising using deep image prior combined with regularization by denoising. IEEE Access. (2021) 9:52378–92. doi: 10.1109/ACCESS.2021.306923625079929

[33] Karakatsanis NA Lodge MA Zhou Y Mhlanga J Chaudhry MA Tahari AK . (2011). “ Dynamic multi-bed FDG PET imaging: feasibility and optimization”, in: 2011 IEEE Nuclear Science Symposium Conference Record (Valencia, Spain: IEEE), 3863–70. doi: 10.1109/NSSMIC.2011.6153735

[34] Karakatsanis NA Lodge MA Tahari AK Zhou Y Wahl RL Rahmim A . Dynamic whole-body PET parametric imaging: I. Concept, acquisition protocol optimization and clinical application. Phys Med Biol. (2013) 58:7391–418. doi: 10.1088/0031-9155/58/20/7391 24080962 PMC3941007

[35] Karakatsanis NA Lodge MA Zhou Y Wahl RL Rahmim A . Dynamic whole-body PET parametric imaging: II. Task-oriented statistical estimation. Phys Med Biol. (2013) 58:7419–45. doi: 10.1088/0031-9155/58/20/7419 24080994 PMC3941010

[36] Fahrni G Karakatsanis NA Di Domenicantonio G Garibotto V Zaidi H . Does whole-body Patlak 18F-FDG PET imaging improve lesion detectability in clinical oncology? Eur Radiol. (2019) 29:4812–21. doi: 10.1007/s00330-018-5966-1 30689031

[37] Karakatsanis NA Zhou Y Lodge MA Casey ME Wahl RL Zaidi H . Generalized whole-body Patlak parametric imaging for enhanced quantification in clinical PET. Phys Med Biol. (2015) 60:8643–73. doi: 10.1088/0031-9155/60/22/8643 26509251 PMC4710061

[38] Karakatsanis NA Casey ME Lodge MA Rahmim A Zaidi H . Whole-body direct 4D parametric PET imaging employing nested generalized Patlak expectation–maximization reconstruction. Phys Med Biol. (2016) 61:5456–85. doi: 10.1088/0031-9155/61/15/5456 27383991 PMC5884686

[39] Zaidi H Karakatsanis N . Towards enhanced PET quantification in clinical oncology. Br J Radiol. (2017) 91:20170508. doi: 10.1259/bjr.20170508 29164924 PMC6049841

